# Efficacy and safety of intravenous thrombolysis versus standard medical management for minor stroke: a systematic review and meta-analysis of RCTs

**DOI:** 10.3389/fneur.2026.1773324

**Published:** 2026-04-29

**Authors:** Xianrong Feng, Yujiao Tang, Baojia Wang, Zhiqing Liu

**Affiliations:** 1 Department of Neurology, Hospital of Chengdu University of Traditional Chinese Medicine, Chengdu, Sichuan, China; 2 Department of Traditional Chinese Medicine, Hospital of Chengdu University of Traditional Chinese Medicine, Chengdu, Sichuan, China; 3 Department of Otorhinolaryngology, Hospital of Chengdu University of Traditional Chinese Medicine, Chengdu, Sichuan, China

**Keywords:** efficacy, intravenous thrombolysis, minor ischemic stroke, safety, standard medical management

## Abstract

**Background:**

The efficacy and safety of intravenous thrombolysis (IVT) compared to standard medical management (SMM) remain unclear in patients with minor ischemic stroke (National Institutes of Health Stroke Scale [NIHSS] ≤ 5). This meta-analysis of randomized controlled trials (RCTs) aimed to synthesize evidence from a direct comparison of these treatments.

**Methods:**

We systematically searched PubMed, Embase, the Cochrane Library, and ClinicalTrials.gov from inception to June 30th, 2025. The primary efficacy outcome was an excellent functional outcome (modified Rankin Scale [mRS] score 0–1) at 90 days. The secondary efficacy outcome was functional independence (mRS score 0–2) at 90 days. Key safety outcomes included symptomatic intracranial hemorrhage (sICH) and 90-day all-cause mortality. Data were analyzed using a random-effects model. This study was registered with PROSPERO (CRD420251089799).

**Results:**

Five RCTs involving 4,361 patients were included. The meta-analysis revealed no significant difference between IVT and SMM in efficacy, both for the primary efficacy outcome (90-day mRS 0–1: OR 0.85, 95% CI 0.72–1.00) or the secondary efficacy outcome (90-day mRS 0–2: OR 0.85, 95% CI 0.63–1.13). Regarding safety, the risk of sICH was significantly higher in the IVT group (OR 4.70, 95% CI 1.76–12.52), whereas no significant difference was found in 90-day all-cause mortality (OR 1.62, 95% CI 0.69–3.79).

**Conclusion:**

In patients with minor ischemic stroke, IVT offers no superior benefit in functional outcomes over SMM but significantly increases sICH risk. Therefore, routine use of IVT should be approached with caution. Future research should identify specific subgroups who might benefit.

**Systematic review registration:**

https://www.crd.york.ac.uk/PROSPERO/view/CRD420251089799.

## 1 Introduction

Minor ischemic stroke (MIS) is commonly defined as an acute ischemic stroke with a National Institutes of Health Stroke Scale (NIHSS) score of ≤5, accounting for more than 50% of all acute ischemic stroke cases (1, 2). Despite the seemingly benign initial symptoms, MIS carries a substantial risk of poor prognosis, with approximately one-third of patients experiencing functional disability within 90 days due to neurological deterioration or recurrent stroke (3–5). While IVT is an established treatment for moderate-to-severe strokes (6, 40), its risk–benefit profile in MIS patients remains unclear (7–9), posing significant challenges in acute management decisions.

Current guidelines recommend IVT for MIS patients with disabling deficits, supported by individual patient data meta-analyses suggesting potential improvements in functional outcomes for this subgroup (6, 10). However, high-quality evidence remains limited and conflicting. The Third International Stroke Trial demonstrated an overall benefit of IVT within 6 hours after onset; however, the benefit diminished in patients with lower baseline NIHSS scores, making the effect in MIS uncertain (11). The Effect of Alteplase vs. Aspirin on Functional Outcome for Patients With Acute Ischemic Stroke and Minor Nondisabling Neurologic Deficits (PRISMS) trial found that, in non-disabling MIS patients, alteplase did not improve 90-day functional outcomes compared to aspirin and significantly increased the risk of symptomatic intracranial hemorrhage (sICH) (12). However, the trial was terminated early due to slow enrollment, limiting the strength of its conclusions. More recent large-scale trials, including Dual Antiplatelet Therapy vs. Alteplase for Patients With Minor Nondisabling Acute Ischemic Stroke (ARAMIS) (13), Prourokinase vs. Standard Care for Patients With Mild Ischemic Stroke (PUMICE) (14), Tenecteplase vs. Standard of Care for Minor Ischaemic Stroke With Proven Occlusion (TEMPO-2) (15), and Effect of Intravenous Urokinase vs. Best Medicine Treatment on Functional Outcome for Patients With Acute Minor Stroke (TRUST) (16), did not demonstrate significant improvements in functional outcomes with IVT, and their safety results were inconsistent. Additionally, observational studies and meta-analyses have reported contradictory findings, with some showing improved outcomes and others emphasizing increased hemorrhagic risks, further complicating clinical decision-making (9, 17–20). At present, comprehensive evidence based exclusively on randomized controlled trials (RCTs) remains limited. Therefore, an updated systematic review of RCT data is urgently needed to clarify the efficacy and safety of IVT in MIS.

Hence, this study aims to conduct a systematic review and meta-analysis of the latest relevant RCTs. By rigorously assessing the risk of bias and performing subgroup analyses, we aim to evaluate the effects of IVT versus standard medical management (SMM) on functional outcomes, mortality, and safety in the overall MIS population and predefined subgroups, thereby providing more robust evidence to guide clinical practice.

## 2 Methods

### 2.1 Study registration

This systematic review and meta-analysis was conducted in accordance with the Preferred Reporting Items for Systematic Reviews and Meta-Analyses (PRISMA) statement to ensure transparent and standardized reporting (21). The study protocol was registered in the International Prospective Register of Systematic Reviews (PROSPERO) registry (CRD420251089799). As this research was a secondary analysis of published studies, ethical approval and patient informed consent were not required.

### 2.2 Eligibility criteria

The inclusion criteria for this study are as follows: The study population consists of adult patients (≥18 years) with acute minor ischemic stroke, defined as a baseline NIHSS score of ≤5. The intervention group receives IVT treatment, which includes alteplase, tenecteplase, urokinase, or prourokinase. The control group must receive standard medical management (predominantly antiplatelet therapy, but discretionary use of anticoagulants was not an exclusion criterion if permitted by trial protocols), with the permissible use of concomitant guideline-recommended secondary prevention treatments. Eligible studies were required to report at least one predefined efficacy or safety outcome, including favorable functional outcomes at 90 days (such as mRS 0–1 or 0–2), sICH, or all-cause mortality.

Exclusion criteria for the study include: (1) non-randomized controlled trials, such as observational studies, case reports, reviews, guidelines, or conference abstracts; (2) studies involving patients who underwent endovascular mechanical thrombectomy or bridging therapy, unless full data on the subgroup receiving only IVT could be independently extracted; (3) studies that did not include an eligible control group; (4) studies where the baseline definition of minor stroke did not align with the criteria of this study; (5) duplicate publications or studies where valid outcome data could not be extracted due to data incompleteness. In cases of data overlap, the most comprehensive publication was included.

### 2.3 Data source and search strategy

We conducted a systematic search of the PubMed, Embase, Cochrane Library, and ClinicalTrials.gov databases. The search encompassed all articles from the inception of each database to June 30th, 2025. The search strategy employed a combination of subject headings and free-text terms, with key search terms including “minor stroke,” “mild stroke,” “acute ischemic stroke,” “thrombolysis,” “intravenous thrombolysis,” “tissue plasminogen activator,” “alteplase,” “tenecteplase,” “urokinase,” “prourokinase,” “antiplatelet,” “aspirin,” “clopidogrel,” and “dual antiplatelet therapy.” The complete and detailed search strategy is provided in the Supplementary Table S1.

Additionally, we manually searched relevant systematic reviews and references from included studies to identify further eligible trials. All records were imported into EndNote, and duplicates were removed before screening.

### 2.4 Study selection

The study selection process was conducted independently by two investigators (XRF and YJT). After initial screening of titles and abstracts and subsequent full-text review, the final included studies were determined. Disagreements were resolved by consensus or through arbitration by a third investigator (ZQL).

### 2.5 Data collection and extraction

Data extraction was independently conducted by two reviewers (XRF and YJT), with all entries cross-checked for accuracy. Any discrepancies were resolved through discussion; if consensus could not be reached, the matter was referred to a third senior reviewer (ZQL) for final adjudication.

A pre-designed, standardized data extraction form was used to systematically collect key information across several domains. This included: fundamental study characteristics (e.g., first author, year of publication, study design, sample size); baseline participant characteristics (e.g., age, sex distribution, major vascular risk factors, baseline NIHSS score, the study-specific definition of minor stroke, and onset-to-treatment time); and details of the interventions, including specific regimens and dosages, along with all reported efficacy and safety outcomes.

### 2.6 Risk of bias and quality of evidence

The risk of bias in included RCTs was assessed using the Cochrane Risk of Bias tool 2 (RoB 2) (22). This tool systematically evaluates five domains: (1) bias arising from the randomization process, (2) bias due to deviations from intended interventions, (3) bias due to missing outcome data, (4) bias in measurement of the outcome, and (5) bias in selection of the reported result. The risk of bias for each domain was judged as “low risk”, “high risk”, or “some concerns”. Subsequently, we evaluated the overall certainty of the evidence for each outcome using the Grading of Recommendations Assessment, Development and Evaluation (GRADE) framework, categorizing it as high, moderate, low, or very low (23). All assessments were conducted independently by two reviewers (XRF and YJT). Any discrepancies were resolved through discussion to achieve consensus. If consensus could not be reached, a senior researcher (ZQL) adjudicated the final decision.

### 2.7 Outcome measurement

The primary efficacy outcome for this meta-analysis was an excellent functional outcome at 90 days, defined as a modified Rankin Scale (mRS) score of 0–1. Secondary efficacy outcomes included a favorable functional outcome at 90 days (mRS score of 0–2). The safety outcomes primarily comprised sICH, all-cause mortality within 90 days. In addition, the following endpoint events were assessed: new vascular events, any adverse events, other bleeding events, and early neurological deterioration (END), and early neurological improvement (ENI).

### 2.8 Statistical analysis

All statistical analyses were conducted using Review Manager (RevMan, version 5.4; The Cochrane Collaboration). A random-effects model was chosen *a priori* to account for potential heterogeneity among the included studies. For dichotomous outcomes, odds ratios (OR) with corresponding 95% confidence intervals (CI) were calculated. Heterogeneity was assessed using Cochran’s *Q* test and the *I*^2^ statistic, with a *Q* test *p*-value < 0.10 or an *I*^2^ value ≥ 50% indicating significant heterogeneity.

Subgroup analyses were preplanned for the primary outcome of 90-day favorable functional outcome (modified Rankin Scale [mRS] score 0–1). These analyses were to be stratified by: (1) patient characteristics, including age, sex, hypertension, diabetes, baseline NIHSS score, and disability; and (2) intervention protocols, including the type of thrombolytic agent (alteplase vs. others) and the antiplatelet strategy in the control group (dual antiplatelet therapy vs. other therapies).

A leave-one-out sensitivity analysis was performed to evaluate the robustness of the results. Given the limited number of included RCTs (<10), a formal assessment of publication bias (e.g., via Egger’s test or funnel plot analysis) and meta-regression were not performed (24). All statistical tests were two-sided, and a *p*-value < 0.05 was considered statistically significant.

## 3 Results

### 3.1 Results of the conducted search

**Figure 1:**
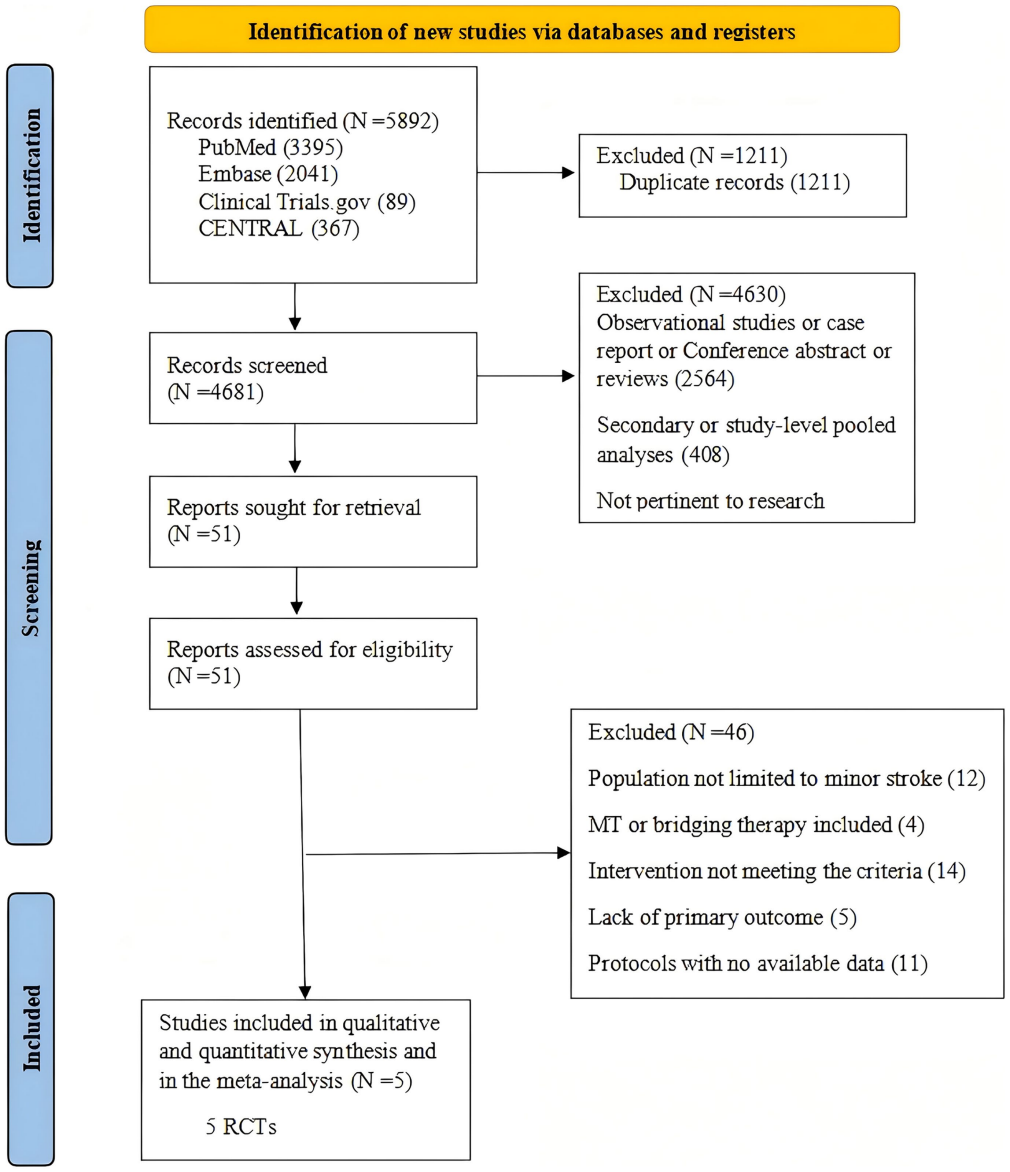
Flowchart of literature search and selection. MT, Mechanical thrombectomy; RCTs, randomized controlled trials.

The systematic literature search initially identified 5,892 records. After removing 1,211 duplicates, 4,681 articles underwent title and abstract screening, which resulted in the exclusion of 4,630 irrelevant studies. The full texts of the remaining 51 articles were subsequently assessed for eligibility. Following a detailed review, 5 RCTs met the inclusion criteria and were included in this systematic review and meta-analysis. The complete study selection process is outlined in the PRISMA flow diagram (Figure 1).

### 3.2 Characteristics of included studies

This study ultimately included 5 RCTs, comprising a total of 4361patients with minor stroke. Of these, 2,164 patients were allocated to the IVT group and 2,197 patients to the SMM group. Published between 2018 and 2025, these trials were conducted across multiple regions, including North America, South America, Europe, Asia, and Oceania. All trials focused on patients with minor ischemic stroke, typically defined by an NIHSS score of ≤5. In the IVT group, alteplase, tenecteplase, urokinase, and prourokinase were used, while control groups received aspirin monotherapy, dual antiplatelet therapy, or best medical therapy. Most trials employed a prospective, randomized, open-label, blinded-endpoint (PROBE) design. The basic characteristics of the included studies are summarized in Table 1.

**Table 1:** Characteristics of included studies.

Study		PRISMS (12)		ARAMIS (13)		PUMICE (14)		TEMPO-2 (15)		TRUST (16)	
Design		Phase 3b, double-blind, double-placebo		Open-label, blinded end point		open-label, blinded end point		parallel group, open label with blinded end point		open-label, blinded end point	
Enrollment time		May 2014–December 2016		October 2018–April 2022		November 2022–December 2023		April 2015–January 2024		October 2020–February 2023	
Country		USA		China		China		Australia, Canada, Austria, Brazil, Finland, Ireland, New Zealand, Spain, Singapore, and the United Kingdom		China	
No. of hospitals		75		38		89		48		25	
Disabling status		Nondisabling		Nondisabling		Both disabling and nondisabling		Both disabling and nondisabling		Both disabling and nondisabling	
Pre-mRS		0–1		0–1		0–1		0–2		0–1	
IVT drug		Alteplase		Alteplase		Prourokinase		Tenecteplase		Urokinase	
Control therapy		SAPT (aspirin 325 mg, single dose, with placebo IVT Alteplase)		DAPT (aspirin 100 mg/day + clopidogrel 75 mg/day (loading 300 mg on day 1) for 12 ± 2 days, then single or dual antiplatelet)		Standard care (DAPT, aspirin, clopidogrel, ticagrelor, anticoagulants or heparin)		Standard care (predominantly SAPT or DAPT)		Best medicine treatment (guideline-based antiplatelets)	
Time window from onset		Within 3 h		Within 4.5 h		Within 4.5 h		Within 12 h		Within 6 h	
Groups		IVT	Control	IVT	Control	IVT	Control	IVT	Control	IVT	Control
No of patients		156	157	350	369	723	723	432	452	503	496
Male, %		77 (49.3)	92 (58.6)	240 (68.6)	256 (69.5)	479 (66.3)	469 (64.9)	244 (56.4)	272 (60.1)	317 (63.0)	311 (62.7)
Age, median (IQR or SD)		62 (14)	61 (13)	64 (56–71)	65 (57–71)	65.9 (57.6–73.2)	65.9 (58.0–72.2)	72 (62–80)	72 (61–79)	62.7 ± 10.7	63.1 ± 10.7
Medical history	Hypertensio*n*, %	126 (81.3)	124 (79.0)	169 (48.3)	211 (57.2)	537 (74.3)	524 (72.5)	265 (61.3)	261 (57.7)	272 (54.1)	253 (51.0)
	Diabetes, %	57 (36.5)	44 (28.0)	86 (24.6)	101 (27.4)	171 (23.7)	192 (26.6)	82 (19.0)	86 (19.0)	75 (14.9)	72 (14.5)
	AF, %	23 (14.7)	17 (10.8)	NA	NA	40 (5.5)	35 (4.8)	91 (21.1)	78 (17.3)	NA	NA
	Ischemic stroke or TIA, %	28 (17.9)	24 (15.3)	79 (22.6)	86 (23.3)	191 (26.4)	200 (27.7)	72 (16.6)	85 (18.8)	151 (30.0)	137 (27.6)
Onset to treatment time, median (IQR)		2.7 (2.2–2.9) hour	2.8 (2.4–3.1) hour	180 (126–225) min	182 (134–230) min	187.0 (142.0–25.0) min	184.0 (138.0–23.0) min	293 (165–453) min	311 (184–495) min	NA	NA
Baseline NIHSS, median (IQR or SD)		2.3 (1.2)	2.0 (1.2)	2 (1–3)	2 (1–3)	3 (2–4)	2 (1–3)	2 (1–3)	2 (1–3)	3 (2–4)	2 (1–4)
Baseline ASPECTS, median (IQR)		10 (7–10)	10 (7–10)	NA	NA	10 (9–10)	10 (9–10)	10 (9–10)	10 (9–10)	NA	NA

RCT, randomized controlled trial; pre-mRS, pre–stroke modified Rankin scale; IVT, intravenous thrombolysis; NA, not applicable/available; NIHSS, National Institutes of Health Stroke Scale; SPECTS, Alberta Stroke Program Early CT Score.

### 3.3 Primary outcomes

#### 3.3.1 mRS scores 0–1 at 90 days

**Figure 2:**
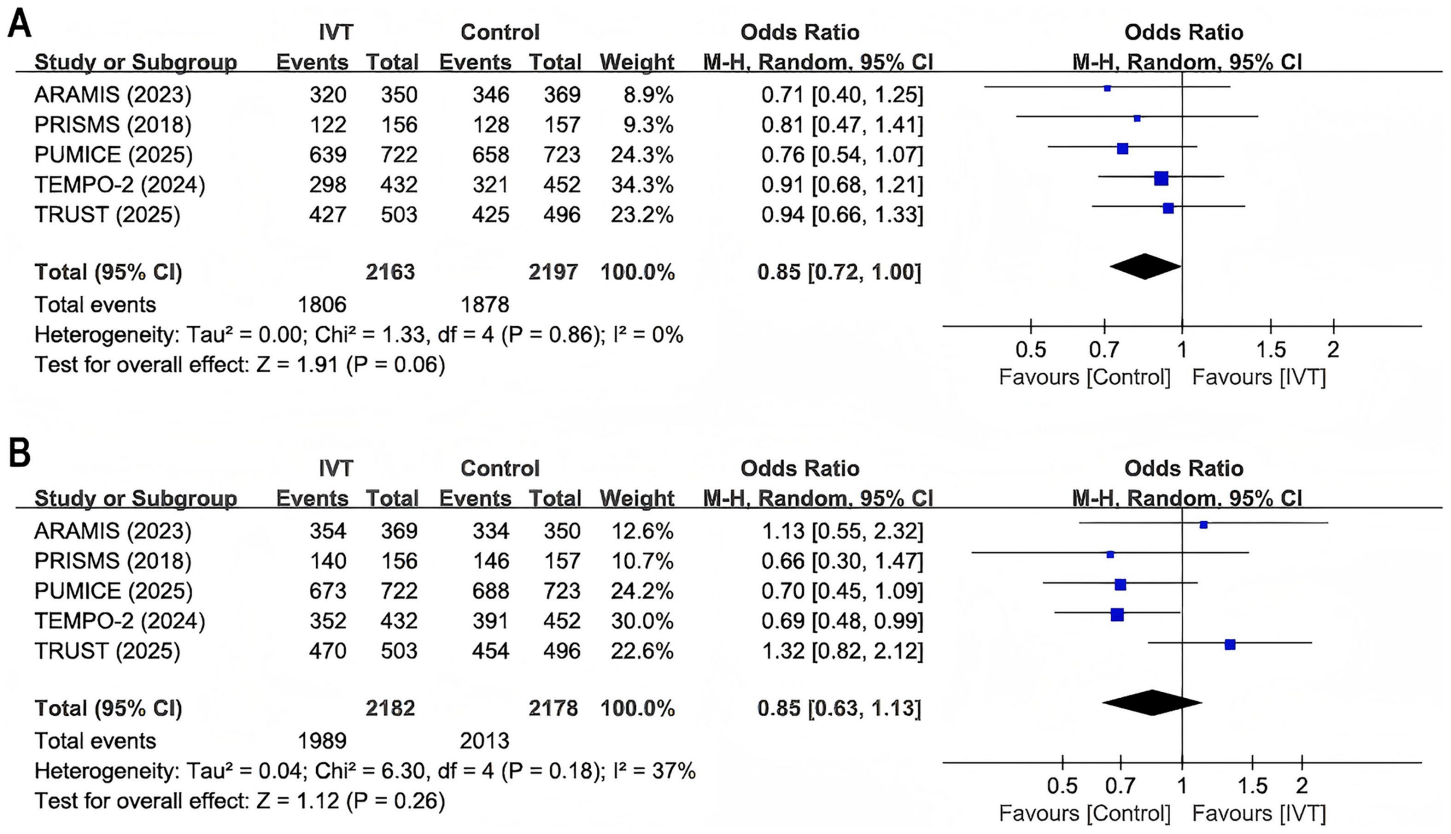
Forest plot for mRS scores 0–1 at 90 days and mRS scores 0–2 at 90 days. **(A)** mRS scores 0–1 at 90 days; **(B)** mRS scores 0–2 at 90 days. ARAMIS = Dual Antiplatelet Therapy vs. Alteplase for Patients With Minor Nondisabling Acute Ischemic Stroke; PRISMS = Effect of Alteplase vs. Aspirin on Functional Outcome for Patients With Acute Ischemic Stroke and Minor Nondisabling Neurologic Deficits; PUMICE = Prourokinase vs. Standard Care for Patients With Mild Ischemic Stroke; TEMPO-2 = Tenecteplase vs. Standard of Care for Minor Ischaemic Stroke With Proven Occlusion; TRUST = Effect of Intravenous Urokinase vs. Best Medicine Treatment on Functional Outcome for Patients With Acute Minor Stroke. IVT, intravenous thrombolysis.

All five included RCTs reported data on the proportion of patients achieving a favorable functional outcome, defined as a modified Rankin Scale (mRS) score of 0–1 at 90 days. The pooled analysis (Figure 2A) revealed no statistically significant difference between the IVT group and the SMM group in the incidence rate of reaching this outcome (OR 0.85, 95% CI 0.72–1.00; *p* = 0.06). No heterogeneity was observed among the studies (*I*^2^ = 0%, *p* = 0.86).

#### 3.3.2 mRS scores 0–2 at 90 days

For the secondary efficacy outcome, functional independence (mRS score 0–2) at 90 days, no significant difference in efficacy was found between the two treatment groups (OR 0.85, 95% CI 0.63–1.13; *p* = 0.26; Figure 2B), with low heterogeneity detected among the studies (*I*^2^ = 37%, *p* = 0.18).

### 3.4 Safety outcomes

#### 3.4.1 sICH

**Figure 3:**
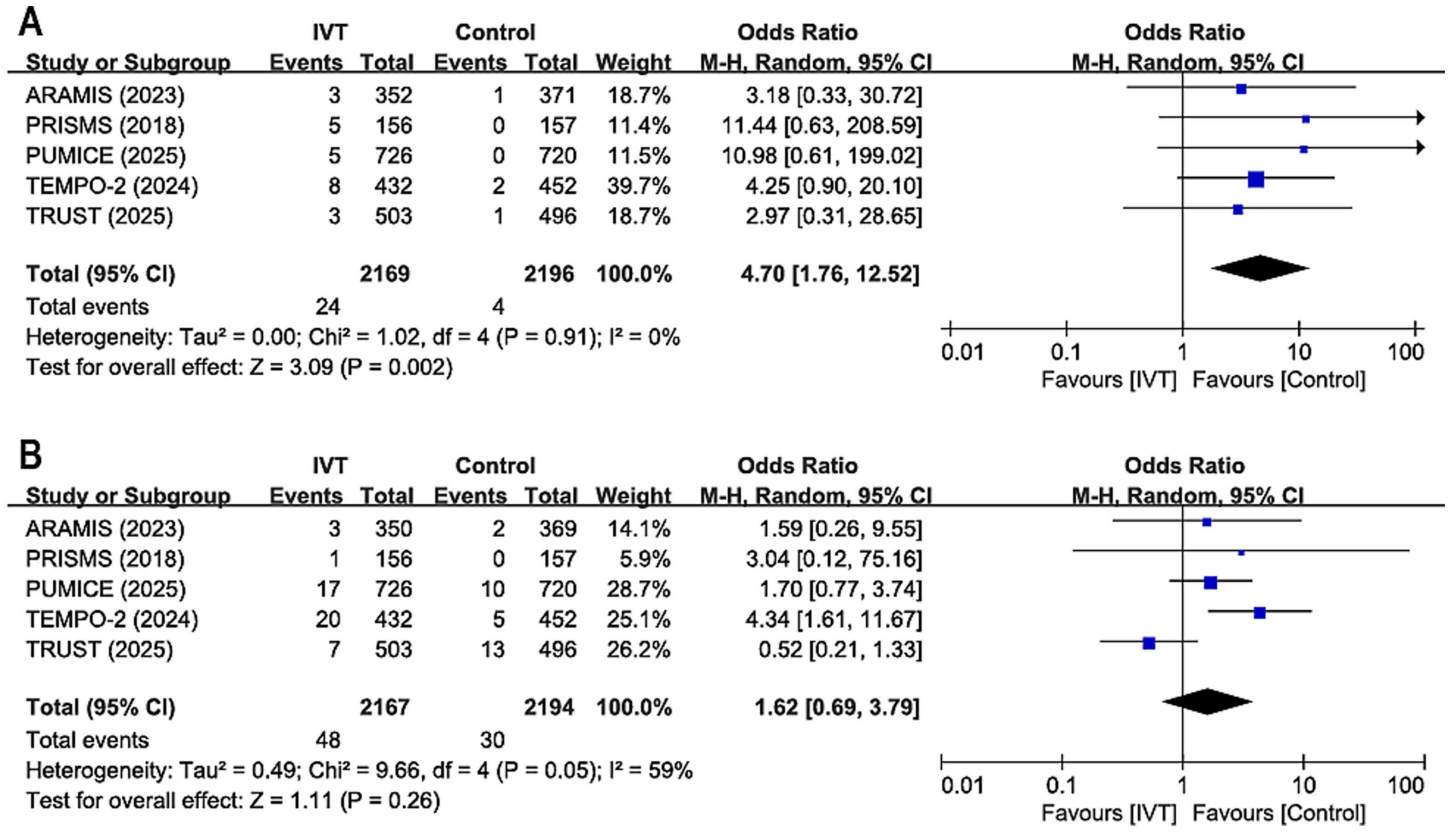
Forest plot for sICH within 36 hours and all-cause mortality at 90 days. **(A)** Symptomatic intracranial hemorrhage within 36 h, **(B)** all-cause mortality at 90 days. ARAMIS = Dual Antiplatelet Therapy vs. Alteplase for Patients With Minor Nondisabling Acute Ischemic Stroke; PRISMS = Effect of Alteplase vs. Aspirin on Functional Outcome for Patients With Acute Ischemic Stroke and Minor Nondisabling Neurologic Deficits; PUMICE = Prourokinase vs. Standard Care for Patients With Mild Ischemic Stroke; TEMPO-2 = Tenecteplase vs. Standard of Care for Minor Ischaemic Stroke With Proven Occlusion; TRUST = Effect of Intravenous Urokinase vs. Best Medicine Treatment on Functional Outcome for Patients With Acute Minor Stroke. sICH, symptomatic intracranial hemorrhage. IVT, intravenous thrombolysis.

All five RCTs provided data on sICH. The analysis revealed that the IVT group significantly increased the risk of sICH compared to the SMM group (OR 4.70, 95% CI 1.76–12.52; *p* = 0.002; Figure 3A), with no statistical heterogeneity observed across studies (*I*^2^ = 0%, *p* = 0.91).

#### 3.4.2 90-day all-cause mortality

The results revealed no significant difference in 90-day all-cause mortality between the IVT and SMM groups (OR 1.62, 95% CI 0.69–3.79; *p* = 0.26), with mild heterogeneity among studies (*I*^2^ = 59%, *p* = 0.05; Figure 3B).

### 3.5 Other endpoint events

**Figure 4:**
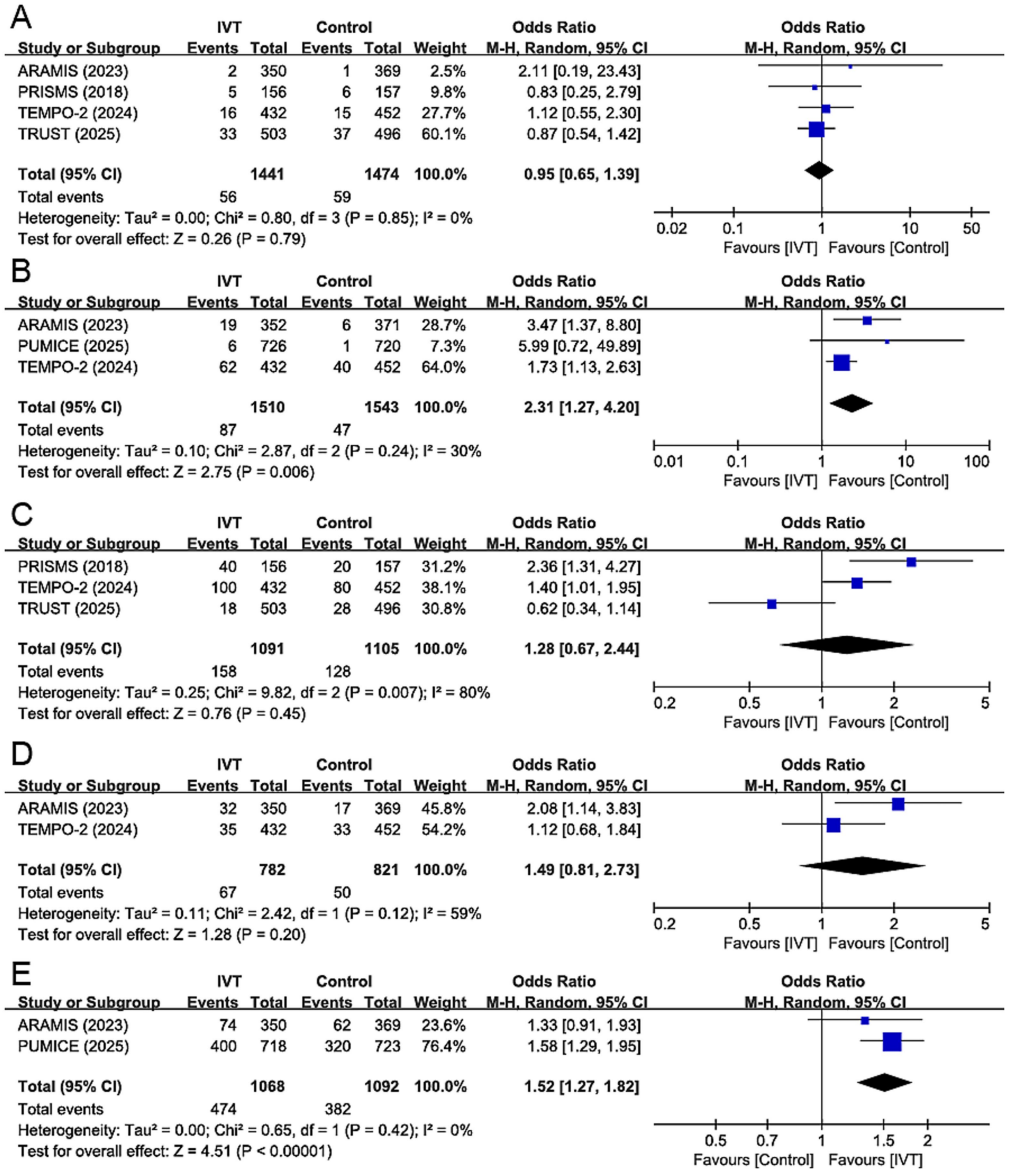
Forest plot for other endpoint events: **(A)** New vascular events; **(B)** other bleeding events; **(C)** adverse events; **(D)** early neurological deterioration; **(E)** early neurological improvement. ARAMIS = Dual Antiplatelet Therapy vs. Alteplase for Patients With Minor Nondisabling Acute Ischemic Stroke; PRISMS = Effect of Alteplase vs. Aspirin on Functional Outcome for Patients With Acute Ischemic Stroke and Minor Nondisabling Neurologic Deficits; PUMICE = Prourokinase vs. Standard Care for Patients With Mild Ischemic Stroke; TEMPO-2 = Tenecteplase vs. Standard of Care for Minor Ischaemic Stroke With Proven Occlusion; TRUST = Effect of Intravenous Urokinase vs. Best Medicine Treatment on Functional Outcome for Patients With Acute Minor Stroke. IVT, intravenous thrombolysis.

This study also evaluated other endpoint events, which are shown in Figure 4. No significant differences were observed between the IVT group and the control group in terms of new vascular events (4 studies), adverse events (3 studies) and END (2 studies), and no inter-study heterogeneities were detected. For other bleeding events (3 studies), the IVT group showed a significantly increased risk (OR 2.31, 95% CI 1.27–4.20, *p* = 0.006), with mild heterogeneity (*I*^2^ = 30%, *p* = 0.24). However, ENI (2 studies), the IVT group had a higher incidence (OR 1.52, 95% CI 1.27–1.82, *p* < 0.001), with no heterogeneity (*I^2^* = 0%, *p* = 0.42).

### 3.6 Risk of bias and GRADE quality of evidence

All 5 included RCTs were assessed as having a low risk of bias. The detailed assessment of each study using the RoB 2 tool is presented in Supplementary Tables S2, S3.

For efficacy outcomes (90-day mRS 0–1 and 0–2) and most key safety outcomes, including symptomatic intracranial hemorrhage, the quality of evidence was rated as “high.” However, due to the moderate to substantial statistical heterogeneity observed, the quality of evidence for 90-day mortality was rated as “moderate.” A detailed summary of the GRADE assessment is provided in Supplementary Table S4.

### 3.7 Sensitivity and subgroup analyses

Leave-one-out sensitivity analyses were performed for all efficacy and primary safety outcomes. Sequential exclusion of each study did not materially alter pooled effect estimates and their statistical significance for the efficacy outcomes (mRS score 0–1 and 0–2 at 90 days) and safety outcomes (sICH and all-cause mortality at 90 days) (Supplementary Table S5). Furthermore, a re-analysis using a fixed-effect model yielded consistent results (Supplementary Table S6).

**Figure 5:**
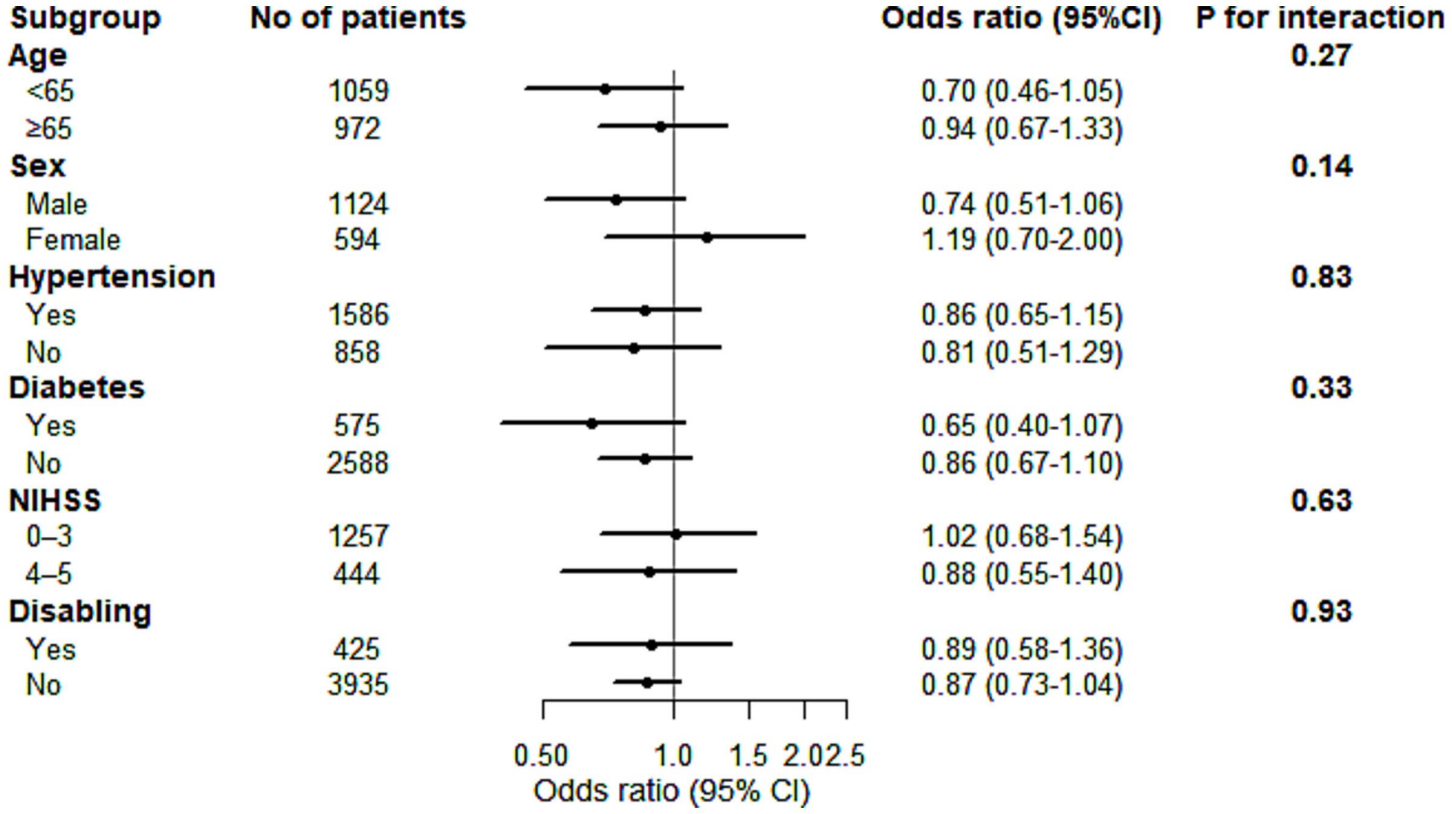
Forest Plot of Age, Sex, Hypertension, Diabetes, Baseline NIHSS Score, and Disabling for 90-Day mRS Scores 0–1.mRS, modified Rankin Scale; NIHSS, National Institutes of Health Stroke Scale; CI, confidence interval. *p value for heterogeneity of subgroup differences.

Prespecified subgroup analyses were conducted for the primary efficacy outcome (mRS 0–1 at 90 days). No significant interaction effects were observed across subgroups stratified by age, sex, hypertension, diabetes, baseline NIHSS score, or disability (all P for interaction > 0.05, Figure 5).

Additionally, further subgroup analyses revealed that neither the type of IVT drug nor the control treatment regimen had a significant interaction effect on the efficacy outcomes (Supplementary Figures S1, S2), the primary safety outcomes (Supplementary Figures S3, S4) or the other endpoint events (Supplementary Figure S5).

## 4 Discussion

This study conducted a systematic review and meta-analysis of five RCTs to compare the efficacy and safety of IVT versus SMM in patients with minor ischemic stroke. The results demonstrated no statistically significant difference between IVT and SMM on functional independence at 90 days (defined as mRS 0–1 or 0–2). In terms of safety, IVT was significantly associated with an increased risk of sICH; although the risk of mortality in the IVT group showed an increasing trend, this difference did not reach statistical significance. Furthermore, the IVT group exhibited a higher incidence of other bleeding events and ENI, while the rates of new vascular events, END, and adverse events were similar between the two groups. These core findings remained consistent across subgroup and sensitivity analyses, further suggesting that this unfavorable risk–benefit profile is not attributable to specific thrombolytic agents or antiplatelet regimens. Therefore, the results of this study indicate that IVT does not provide clear functional benefits in the minor stroke population and may be associated with greater safety risks, indicating that its application in clinical practice warrants careful consideration.

This meta-analysis of five RCTs demonstrates that for patients with mild ischemic stroke, IVT does not offer a significant benefit over SMM in terms of functional outcomes at 90 days. This primary finding provides relatively high-quality evidence to address the ongoing clinical controversy in this field and challenges current guidelines that recommend thrombolysis for patients with “mild but disabling” deficits (6). The conclusion that IVT fails to improve functional outcomes is consistent with results from pivotal recent RCTs, including PRISMS and ARAMIS (12, 13), and is likely influenced by multiple factors. Specifically, first, the inherently favorable natural course of mild stroke and the limited ischemic penumbra restrict the potential benefit of reperfusion therapy (25). Second, the intrinsic bleeding risk of IVT, especially the significantly increased risk of sICH, results in an unfavorable risk–benefit balance when weighed against its limited efficacy (26). Third, advances in the standard care of the control group, including the adoption of more effective antiplatelet regimens such as dual antiplatelet therapy (DAPT), have already led to significant improvements in patient prognosis (27, 28), narrowing the therapeutic window for any additional benefit from IVT. Although earlier observational studies suggested potential benefits of IVT (17, 29–32), these discrepancies are likely attributable to selection biases, variations in stroke severity definitions, and historical limitations in control group treatments (33, 34).

The robustness of the primary outcome was further confirmed through subgroup and sensitivity analyses. These analyses revealed no advantage of IVT in improving functional outcomes, regardless of the thrombolytic agent used (e.g., alteplase vs. newer-generation thrombolytics) or the antiplatelet regimen in the control group. Notably, even in the subgroup of patients with “disabling” symptoms—those most commonly considered for thrombolysis in clinical practice—IVT did not demonstrate a significant treatment effect. Furthermore, key clinical factors such as baseline NIHSS score, age, and history of diabetes did not modify the primary result. In conclusion, the synthesized evidence from this meta-analysis suggests that, in the context of standard medical management, IVT may not provide additional 90-day functional benefits for the broader population of patients with mild ischemic stroke.

This meta-analysis highlights the clear safety risks associated with IVT in patients with mild stroke, with the key finding that IVT is significantly linked to an increased risk of sICH. This conclusion is highly consistent with results from pivotal RCTs and previous meta-analyses (13, 15, 19, 35) but contrasts with findings that suggested IVT is relatively safe (9, 14, 16). The discrepancy likely arises from inherent selection biases in these studies, where clinicians tend to administer thrombolysis to patients with lower baseline risks, thus underestimating the true risks of IVT.

There was no significant difference in mortality between the groups, which aligns with the conclusions of two recent meta-analyses of retrospective studies (36, 37). Although statistical heterogeneity was present in the pooled effect estimate for mortality—potentially stemming from differences in inclusion/exclusion criteria and treatment protocols across trials—sensitivity analyses confirmed the robustness of the results. Additionally, this study identified a complex pattern of outcomes: IVT was associated with a higher risk of other bleeding events, but also with an increased rate of ENI. This suggests that while thrombolysis may induce short-term neurological recovery, such early gains did not translate into long-term functional independence and might be achieved at the cost of a significantly elevated hemorrhagic risk.

Several recent meta-analyses, which included both RCTs and observational studies, have suggested that IVT does not significantly improve functional independence in minor stroke (9, 35, 38), Our study, by strictly limiting inclusion to RCTs, provides a complementary high-level evidence base. Compared with a recent RCT-based meta-analysis (39), our findings align in demonstrating that IVT does not improve mRS 0–1 outcomes and increases the risk of sICH. However, we diverge significantly regarding mRS 0–2 and 90-day mortality. While Doheim et al. associated IVT with poorer functional independence and higher mortality, our analysis attenuated these pooled estimates and shifted the overall consensus to neutral by incorporating the large-scale TRUST study (*N* = 999). Specifically, in TRUST, IVT yielded numerically higher rates of mRS 0–2 (93.4% vs. 91.5%) and lower mortality (1.4% vs. 2.6%). We propose that this discrepancy stems from the unique pharmacological profile of the urokinase used in TRUST. Unlike the fibrin-specific agents typically employed in other trials, the low-dose (1,000,000 U), non-fibrin-specific urokinase regimen may offer a milder thrombolytic effect coupled with an improved safety profile. Consequently, this narrows the incremental benefit of IVT relative to optimal medical managemen without increasing mortality.

This more robust evidence, combined with findings from multiple pivotal RCTs, is driving a paradigm shift in the management of mild stroke: clinical focus is moving from routine IVT to optimized medical management, with early dual antiplatelet therapy (DAPT) as the standard approach, as established by studies like CHANCE and POINT (27, 28). This shift also advances the exploration of the “precision medicine” hypothesis—whether IVT can benefit specific subgroups with documented vascular occlusion. This hypothesis was directly tested in the landmark TEMPO-2 trial included in our analysis, but its results were neutral (15), strongly reinforcing our overall conclusion that the clinical net benefit of IVT remains unproven, even in imaging-selected “high-risk” subgroups. Nonetheless, the exploration of IVT in minor stroke continues. The results of an ongoing clinical trial, Teneteplase Reperfusion Therapy in Acute Ischemic Cerebrovascular Events-IV (NCT06414499), will provide further guidance for IVT treatment decisions in this patient population.

## 5 Limitations

This study has several limitations. First, the final analysis included only five RCTs, and the limited number of events for certain safety endpoints reduced the statistical power of subgroup analyses. Consequently, these results should be interpreted with caution. Additionally, the absence of individual patient data limited our ability to explore potential effect modifiers, such as imaging characteristics (e.g., large vessel occlusion). Second, the generalizability of our findings is influenced by two aspects. On the one hand, treatment protocols varied considerably across the included trials, encompassing divergent thrombolytic agents in the IVT groups, as well as different standard medical management regimens in the control groups (e.g., varying aspirin doses, single vs. dual antiplatelet therapy) and, in the case of the PUMICE trial, discretionary use of anticoagulants. This treatment heterogeneity may introduce confounding factors when assessing the true net benefit of IVT. On the other hand, the definitions of outcome measures were inconsistent across studies: not only did the criteria for determining “disabling” stroke differ, but the sICH definitions also varied, introducing clinical heterogeneity in safety outcomes. We recommend that future stroke trials adopt a standardized sICH definition to improve cross-study comparability. Third, the external validity of our conclusions may be limited by ethnic homogeneity (75% of patients were Chinese, with a relatively small Western sample) and the exclusion of patients receiving endovascular therapy. Thus, caution is needed when extrapolating these findings to other ethnic groups or to scenarios involving aggressive reperfusion for large vessel occlusion. Therefore, large-scale RCTs across diverse ethnic and geographic populations, combined with advanced imaging techniques for precise patient stratification, as well as an individual patient data meta-analysis, are urgently needed. These efforts will provide the definitive evidence necessary to guide personalized treatment approaches for mild stroke.

## 6 Conclusion

In conclusion, this meta-analysis synthesizes the highest level of current evidence, demonstrating that IVT does not provide additional benefits in 90-day functional outcomes for the broader population of patients with mild ischemic stroke. On the contrary, it significantly increases the risk of sICH. Therefore, a cautious approach to the application of IVT is imperative in current clinical practice, with individualized risk assessment being the cornerstone of therapeutic decision-making.

## Data Availability

The original contributions presented in the study are included in the article/Supplementary material, further inquiries can be directed to the corresponding author.
