# Supplementary material for: Efficacy and safety of intravenous thrombolysis versus standard medical management for minor stroke: a systematic review and meta-analysis of RCTs
Source: Front Neurol. 2026 Apr 29;17:1773324. doi: 10.3389/fneur.2026.1773324 (PMC13167584; doi:10.3389/fneur.2026.1773324)
Supplement: Supplementary file 1 [file Data_Sheet_1.PDF]

Supplementary Material

Table of Contents

sFigure 1. Forest Plot of IV alteplase vs. IV non-alteplase (tenecteplase, prourokinase and urokinase) for efficacy outcomes. A, 90-day mRS 0-1; B, 90-day mRS 0-2. 2

sFigure 2. Forest Plot of control regimen and overall resultsfor efficacy outcomes , assessing the impact of control strategies in minor acute ischemic stroke patients treated with IVT. A, 90-day mRS 0-1; B, 90-day mRS 0-2. 3

sFigure 3. Forest Plot of IV alteplase vs. IV non-alteplase (tenecteplase, prourokinase and urokinase) for safety outcomes. A, sICH within 36h; B, all-cause mortality at 90 days.. 4

sFigure 4. Forest Plot of control regimen and overall results for safety outcomes, assessing the impact of control strategies in minor acute ischemic stroke patients treated with IVT. A, sICH within 36h; B, all-cause mortality at 90 days. 5

sFigure 5. Forest Plot of IVT type and control regimen for new vascular events. A, IV alteplase vs.IV non-alteplase (tenecteplase, prourokinase and urokinase), B, control regimen (ASA, DAPT and BMT. 6

sTable 1. Search strategies in (A) PubMed, (B) Embase, (C) Cochrane Central Register of Controlled Trials, and (D) ClinicalTrials.gov 7

sTable 2. Details on quality assessment of the included randomized controlled trials using RoB2. 9

sTable 3. Quality assessment of included randomized controlled trials 11

sTable 4. The Grading of Recommendations, Assessment, Development, and Evaluation (GRADE) system for the outcomes of interest 12

sTable 5. Sensitivity analyses by excluding one trial at a time from the pooled estimates. 13

sTable 6. Analyses using random-effect models for the outcomes of interest. 14

sFigure 1. Forest Plot of IV alteplase vs. IV non-alteplase (tenecteplase, prourokinase and urokinase) for efficacy outcomes. A, 90-day mRS 0-1; B, 90-day mRS 0-2.

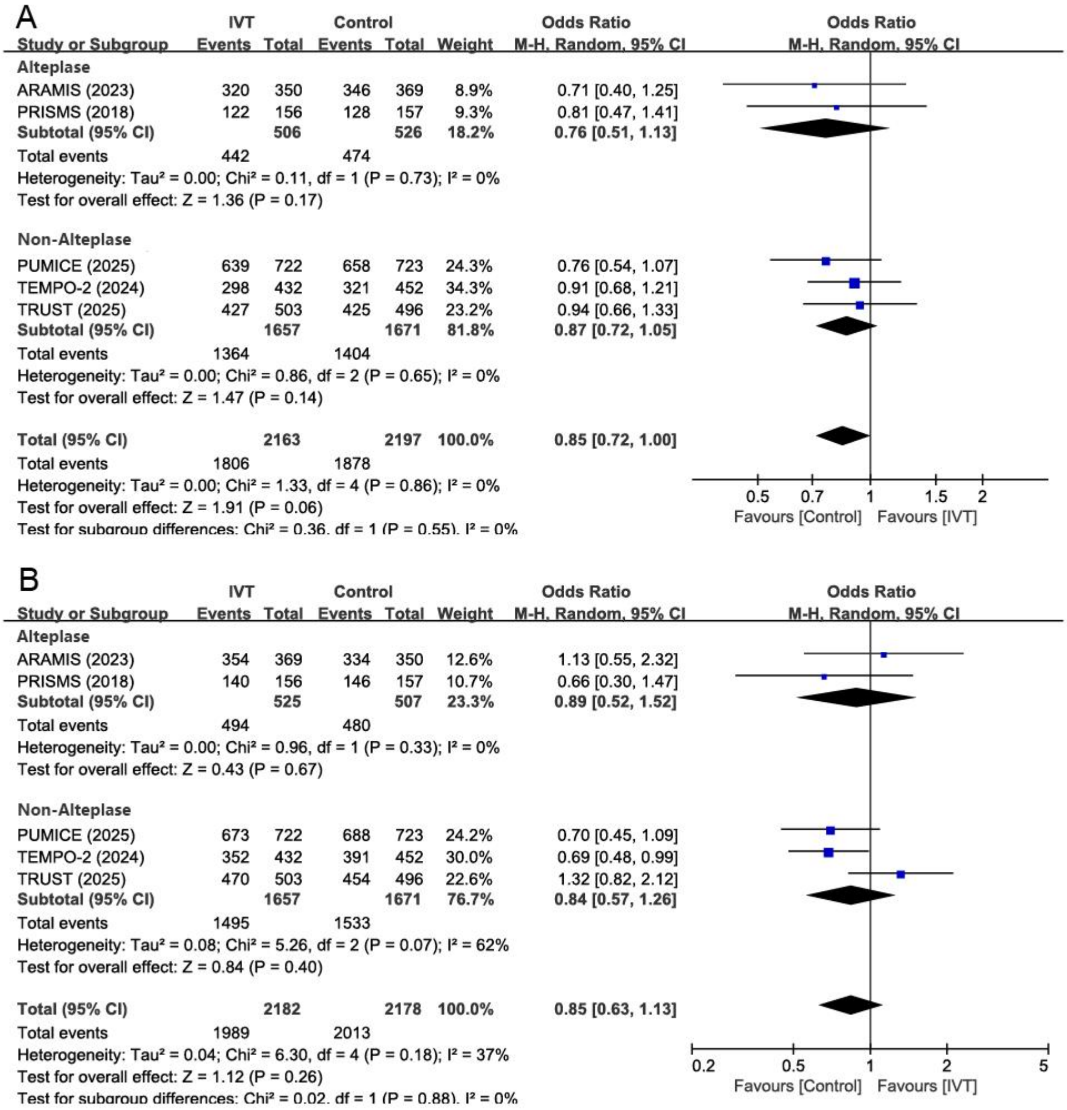

sFigure 2. Forest Plot of control regimen and overall results for efficacy outcomes, assessing the impact of control strategies in minor acute ischemic stroke patients treated with IVT. A, 90-day mRS 0-1; B, 90-day mRS 0-2.

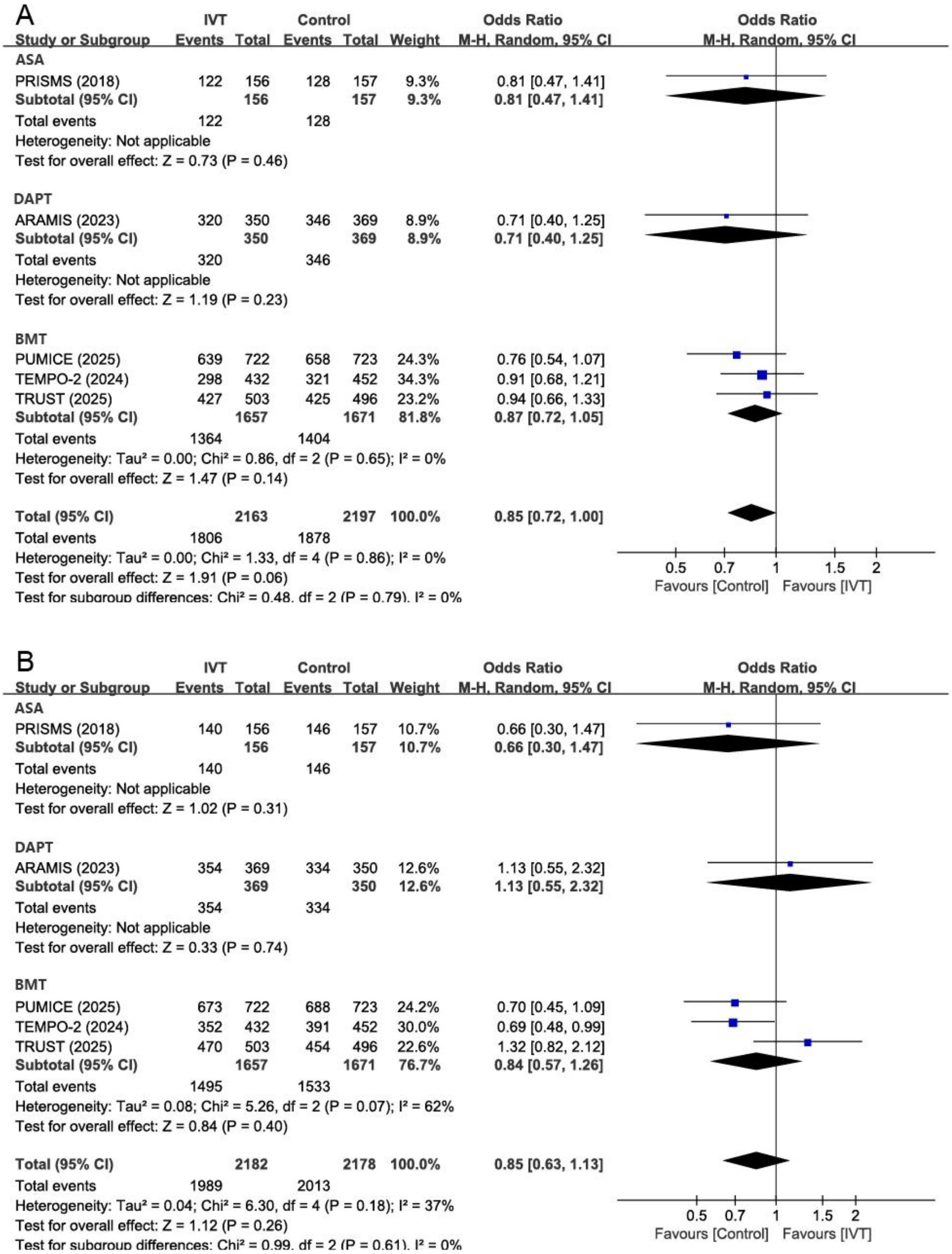

sFigure 3. Forest Plot of IV alteplase vs. IV non-alteplase (tenecteplase, prourokinase and urokinase) for safety outcomes. A, sICH within 36h; B, all-cause mortality at 90 days.

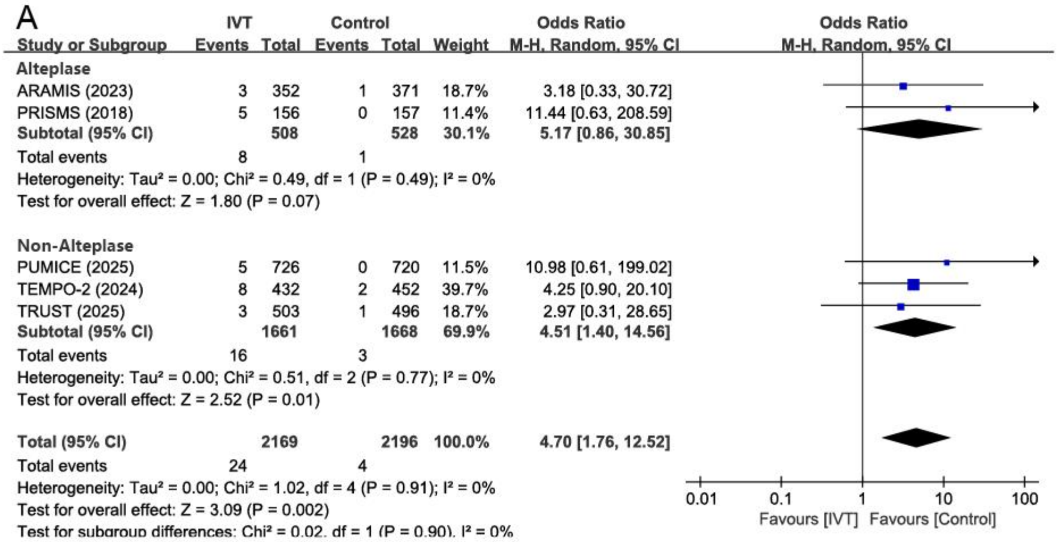

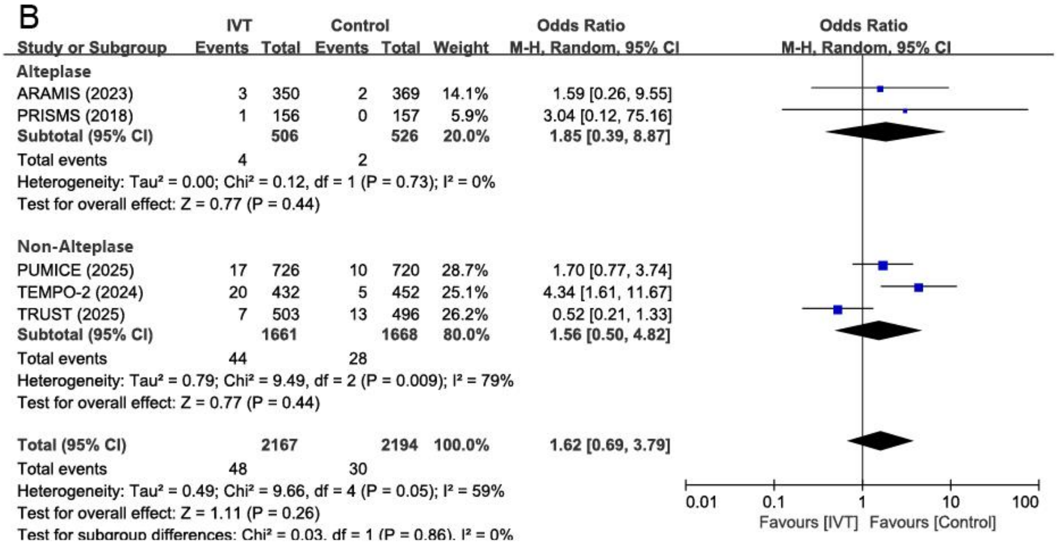

sFigure 4. Forest Plot of control regimen and overall results for safety outcomes, assessing the impact of control strategies in minor acute ischemic stroke patients treated with IVT. A, sICH within 36h; B, all-cause mortality at 90 days.

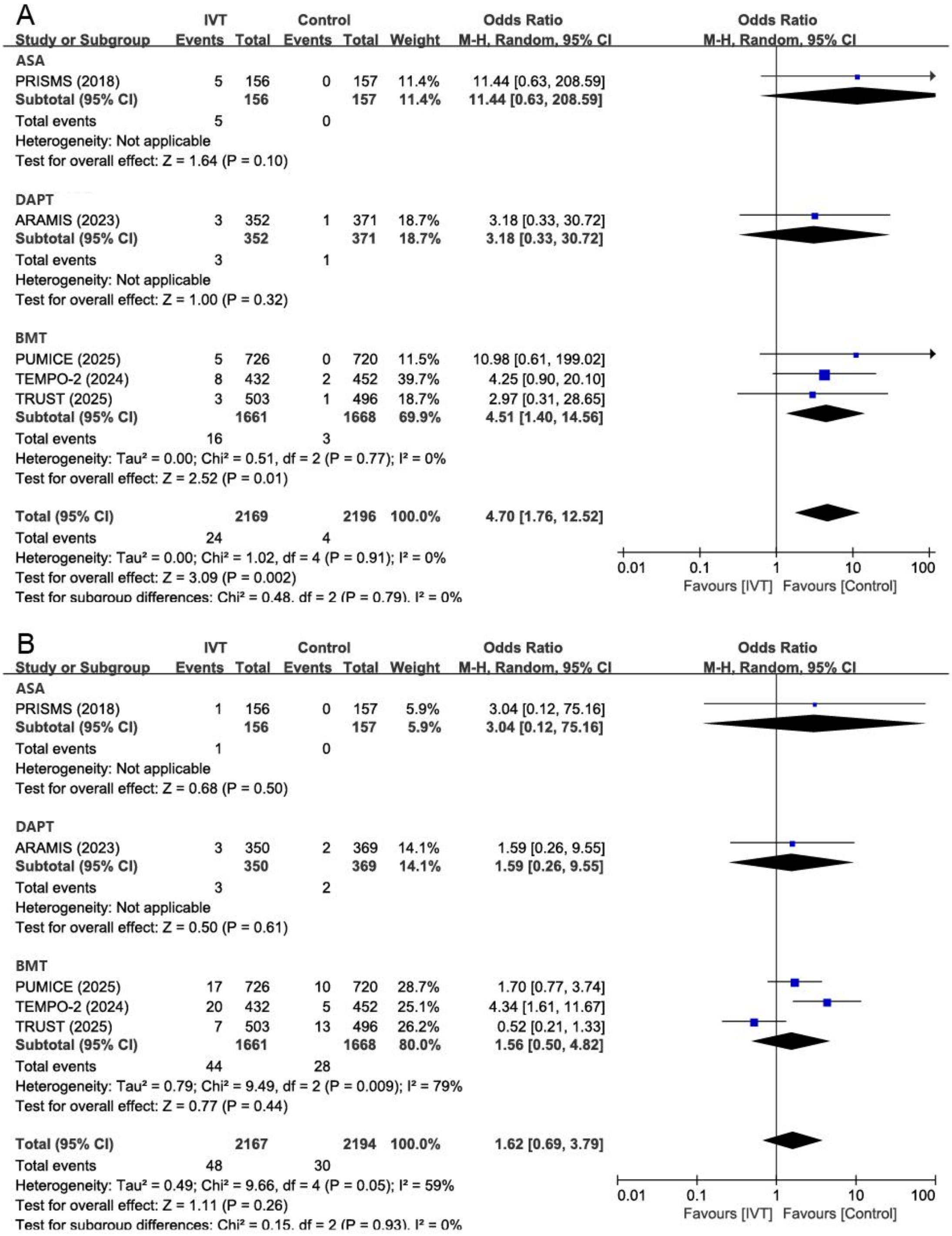

sFigure 5. Forest Plot of IVT type and control regimen for new vascular events. A, IV alteplase vs.IV non-alteplase (tenecteplase, prourokinase and urokinase), B, control regimen (ASA, DAPT and BMT).

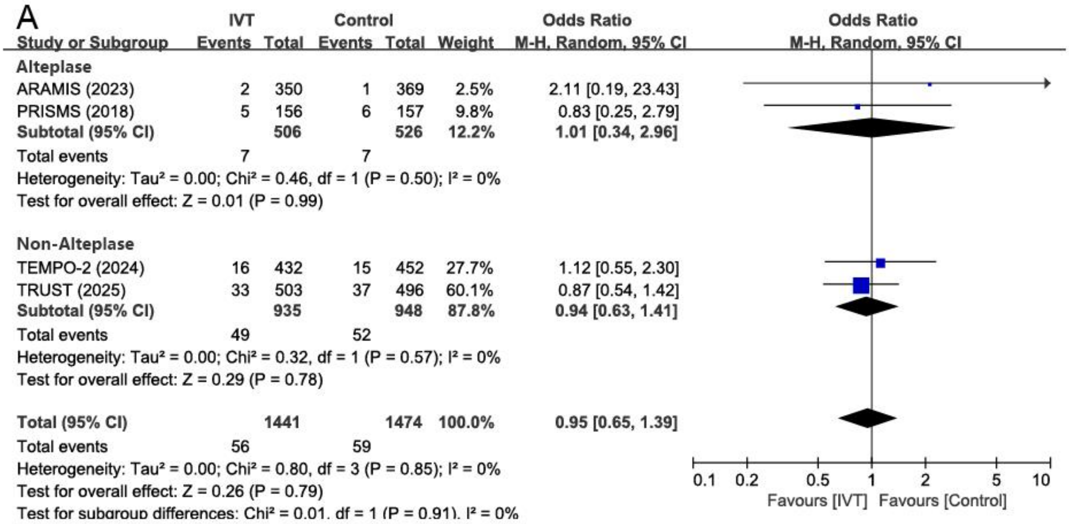

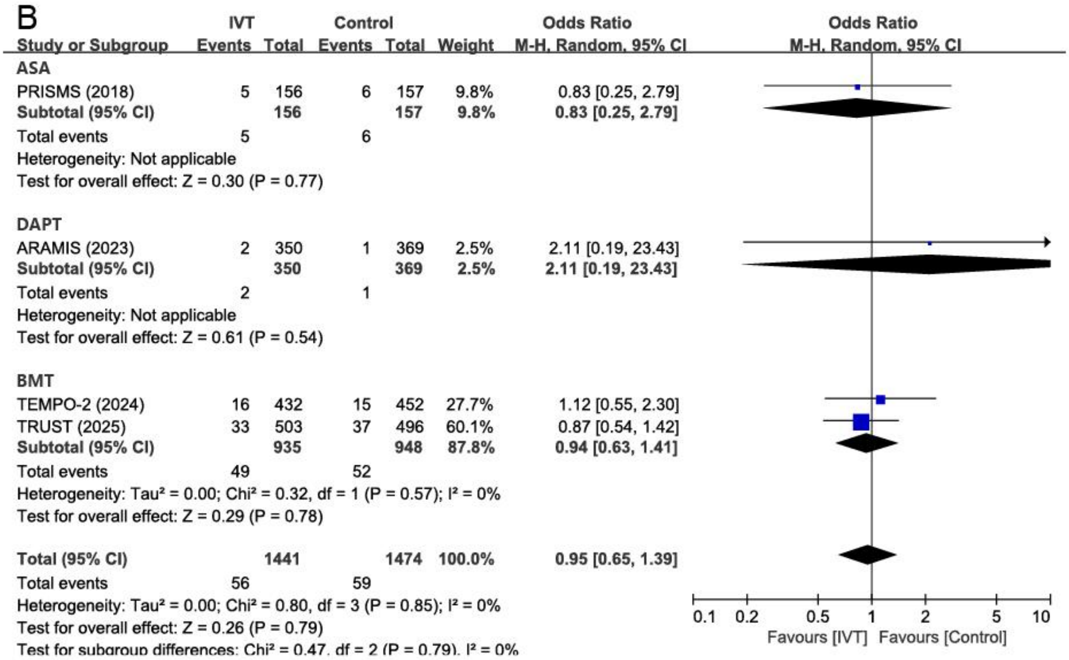

sTable 1. Search strategies in (A) PubMed, (B) Embase, (C) Cochrane Central Register of Controlled Trials, and (D) Clinical Trials.gov.

**(A) PubMed 3395**
#1 "Stroke"[Mesh] OR "Ischemic Stroke"[Mesh] OR "Brain Ischemia"[Mesh] OR stroke[Title/Abstract] OR cerebral ischemia[Title/Abstract] OR brain ischemia[Title/Abstract] OR cerebrovascular accident[Title/Abstract] OR CVA[Title/Abstract]
#2 "minor stroke"[Title/Abstract] OR "mild stroke"[Title/Abstract] OR "mild ischemic stroke"[Title/Abstract] OR "minor ischemic stroke"[Title/Abstract] OR "nondisabling stroke"[Title/Abstract] OR "non-disabling stroke"[Title/Abstract] OR "rapidly improving symptoms"[Title/Abstract] OR mild[Title/Abstract] OR minor[Title/Abstract] OR low NIHSS[Title/Abstract] OR "NIHSS 0-5"[Title/Abstract] OR "NIHSS ≤5"[Title/Abstract] OR "NIHSS <6"[Title/Abstract] OR "National Institutes of Health Stroke Scale"[Title/Abstract]
#3 "Fibrinolytic Therapy"[Mesh] OR "Thrombolytic Therapy"[Mesh] OR "Tissue Plasminogen Activator"[Mesh] OR "Tenecteplase"[Mesh] OR "Urokinase-Type Plasminogen Activator"[Mesh] OR thrombolysis[Title/Abstract] OR thrombolytic[Title/Abstract] OR fibrinolytic[Title/Abstract] OR thromboly*[Title/Abstract] OR "Intravenous Thrombolysis"[Title/Abstract] OR IVT[Title/Abstract] OR "Fibrinolytic Therapy"[Title/Abstract] OR tPA[Title/Abstract] OR "t-PA"[Title/Abstract] OR rtPA[Title/Abstract] OR "rt-PA"[Title/Abstract] OR alteplase[Title/Abstract] OR tenecteplase[Title/Abstract] OR TNK[Title/Abstract] OR urokinase[Title/Abstract] OR prourokinase[Title/Abstract]
#4 "Platelet Aggregation Inhibitors"[Mesh] OR "Aspirin"[Mesh] OR "Clopidogrel"[Mesh] OR antiplatelet[Title/Abstract] OR "anti-platelet"[Title/Abstract] OR "Platelet aggregation inhibitor"[Title/Abstract] OR aspirin[Title/Abstract] OR acetylsalicylic acid[Title/Abstract] OR ASA[Title/Abstract] OR clopidogrel[Title/Abstract] OR "Dual antiplatelet therapy"[Title/Abstract] OR DAPT[Title/Abstract] OR "antiplatelet therapy"[Title/Abstract]
#5 #1 AND #2 AND (#3 OR #4)
**(B) Embase 2041**
#1 'cerebrovascular accident'/exp OR 'ischemic stroke'/exp OR 'brain ischemia'/exp
#2 'stroke':ab,ti OR 'cerebral ischemia':ab,ti OR 'brain ischemia':ab,ti OR 'cerebrovascular accident':ab,ti OR 'cva':ab,ti
#3 (#1 OR #2)
#4 'minor stroke':ab,ti OR 'mild stroke':ab,ti OR 'mild ischemic stroke':ab,ti OR 'minor ischemic stroke':ab,ti OR 'nondisabling stroke':ab,ti OR 'non-disabling stroke':ab,ti OR 'rapidly improving symptoms':ab,ti OR 'low nihss':ab,ti OR 'nihss 0-5':ab,ti OR 'nihss 5 or less':ab,ti OR 'nihss up to 5':ab,ti OR 'nihss less than 6':ab,ti
#5 'fibrinolytic therapy'/exp OR 'thrombolytic therapy'/exp OR 'tissue plasminogen activator'/exp OR 'tenecteplase'/exp OR 'urokinase'/exp
#6 'thrombolysis':ab,ti OR 'thrombolytic':ab,ti OR 'fibrinolytic':ab,ti OR 'thromboly*':ab,ti OR 'intravenous thrombolysis':ab,ti OR 'ivt':ab,ti
#7 'tpa':ab,ti OR 't-pa':ab,ti OR 'rtpa':ab,ti OR 'rt-pa':ab,ti OR 'alteplase':ab,ti OR 'tenecteplase':ab,ti OR 'tnk':ab,ti OR 'urokinase':ab,ti OR 'prourokinase':ab,ti
#8 (#5 OR #6 OR #7)
#9 'antiplatelet agent'/exp OR 'aspirin'/exp OR 'clopidogrel'/exp
#10 'antiplatelet':ab,ti OR 'anti-platelet':ab,ti OR 'platelet aggregation inhibitor':ab,ti OR

'aspirin':ab,ti OR 'acetylsalicylic acid':ab,ti OR 'asa':ab,ti OR 'clopidogrel':ab,ti OR 'dual antiplatelet therapy':ab,ti OR 'dapt':ab,ti<br>#11 (#9 OR #10)<br>#12 (#3 AND #4 AND (#8 OR #11))
**(C) Cochrane Central Register of Controlled Trials 367**
("minor stroke" OR "mild stroke" OR "nondisabling stroke" OR "low NIHSS" OR "non-disabling stroke") AND ("thrombolysis" OR "thrombolytic therapy" OR "fibrinolytic" OR "alteplase" OR "tPA" OR "tenecteplase" OR "urokinase" OR "prourokinase" OR "antiplatelet" OR "aspirin" OR "clopidogrel" OR "DAPT" OR "dual antiplatelet therapy")
**(D) ClinicalTrials.gov 89**
("minor stroke" OR "mild stroke" OR "acute ischemic stroke" OR "NIHSS" OR "low NIHSS") AND ("intravenous thrombolysis" OR IVT OR thrombolysis OR alteplase OR tenecteplase OR urokinase OR "tissue plasminogen activator" OR tPA OR rtPA) AND (antiplatelet OR aspirin OR clopidogrel OR "dual antiplatelet therapy" OR DAPT)

sTable 2. Details on quality assessment of the included randomized controlled trials using RoB2.

Bias Domain and Signaling Question	ARAMIS (2023)	**PRISMS (2018)**	**PUMICE (2025)**	**TEMPO-2 (2024)**	**TRUST (2025)**
**1. Bias Arising from the Randomization Process**					
1.1 Was the allocation sequence random?	Yes	Yes	Yes	Yes	Yes
1.2 Was the allocation sequence concealed until participants were enrolled and assigned to interventions?	Yes	Yes	Yes	Yes	Yes
1.3 Did baseline differences between intervention groups suggest a problem with the randomization process?	No	No	No	No	No
Risk-of-Bias Judgment	Low	Low	Low	Low	Low
Optional: What is the predicted direction of bias arising from the randomization process?	Neutral	Neutral	Neutral	Neutral	Neutral
**2. Bias Due to Deviations from Intended Interventions**					
2.1 Were participants aware of their assigned intervention during the trial?	Yes	Yes	Yes	Yes	Yes
2.2 Were carers and people delivering the interventions aware of participants' assigned intervention during the trial?	Yes	No	Yes	Yes	Yes
2.3 If Yes/Probably Yes/No Information to 2.1 or 2.2: Were there deviations from the intended intervention that arose because of the trial context?	No	No	No	No	No
2.4 If Yes/Probably Yes to 2.3: Were these deviations likely to have affected the outcome?	No	No	No	No	No
2.5 If Yes/Probably Yes to 2.4: Were these deviations from the intended intervention balanced between groups?	Yes	Yes	Yes	Yes	Yes
2.6 Was an appropriate analysis used to estimate the effect of assignment to intervention?	Yes	Yes	Yes	Yes	Yes
2.7 If No/Probably No/No Information to 2.6: Was there potential for a substantial impact (on the result) of the failure to analyze participants in the group to which they were randomized?	No	No	No	No	No
Risk-of-Bias Judgment	Low	Low	Low	Low	Low
Optional: What is the predicted direction of bias due to deviations from intended interventions?	Neutral	Neutral	Neutral	Neutral	Neutral
**3. Bias Due to Missing Outcome Data**					
3.1 Were data for this outcome available for all, or nearly all, participants randomized?	Yes	Yes	Yes	Yes	Yes
3.2 If No/Probably No/ No Information to 3.1: Is there evidence that the result was not biased by missing outcome data?	Yes	Yes	Yes	Yes	Yes
3.3 If No/Probably No to 3.2: Could missingness in the outcome depend on its true value?	No	No	No	No	No
3.4 If Yes/Probably Yes/ No Information to 3.3: Is it likely that missingness in the outcome depended on its true value?	No	No	No	No	No
Risk-of-Bias Judgment	Low	Low	Low	Low	Low
Optional: What is the predicted direction of bias due to missing outcome data?	Neutral	Neutral	Neutral	Neutral	Neutral

**4. Bias in Measurement of the Outcome**					
4.1 Was the method of measuring the outcome inappropriate?	No	No	No	No	No
4.2 Could measurement or ascertainment of the outcome have differed between intervention groups?	No	No	No	No	No
4.3 If No/Probably No/ No Information to 4.1 and 4.2: Were outcome assessors aware of the intervention received by study participants?	No	No [a]	No	No	No
4.4 If Yes/Probably Yes/ No Information to 4.3: Could assessment of the outcome have been influenced by knowledge of intervention received?	No	No	No	No	No
4.5 If Yes/Probably Yes/ No Information to 4.4: Is it likely that the assessment of the outcome was influenced by knowledge of the intervention received?	No	No	No	No	No
Risk-of-Bias Judgment	Low	Low	Low	Low	Low
Optional: What is the predicted direction of bias in the measurement ofthe outcome?	Neutral	Neutral	Neutral	Neutral	Neutral
**5. Bias in Selection of the Reported Result**					
5.1 Were the data that produced this result analyzed in accordance with a prespecified analysis plan that was finalized before unblinded outcome data were available for analysis?	Yes	Yes	Yes	Yes	Yes
Is the numerical result being assessed likely to have been selected, on the basis of the results, from:					
5.2 ... multiple eligible outcome measurements (e.g., scales, definitions, time points) within the outcome domain?	No	No	No	No	No
5.3 … multiple eligible analyses of the data?	No	No	No	No	No
Risk-of-Bias Judgment	Low	Low	Low	Low	Low
Optional: What is the predicted direction of bias due to the selection of the reported results?	Neutral	Neutral	Neutral	Neutral	Neutral
**Overall Bias**					
Risk-of-Bias Judgment	Low	Low	Low	Low	Low
Optional: What is the overall predicted direction of bias for this outcome?	Neutral	Neutral	Neutral	Neutral	Neutral

sTable 3. Quality assessment of included randomized controlled trials

Trial	Year	Blinding	Event adjudication	Overall risk of bias (Low risk ✅ ; High risk ×	
ARAMIS	2023	Open-label	Blinded	1) Randomization process ✅<br>2) Deviations from intended interventions ✅<br>3) Missing outcome data ✅<br>4) Measurement of the outcome ✅<br>5) Selection of the reported result ✅	✅
PRISMS	2018	double -blind,	Blinded	1) Randomization process ✅<br>2) Deviations from intended interventions ✅<br>3) Missing outcome data ✅<br>4) Measurement of the outcome ✅<br>5) Selection of the reported result ✅	✅
PUMICE	2025	Open-label	Blinded	1) Randomization process ✅<br>2) Deviations from intended interventions ✅<br>3) Missing outcome data ✅<br>4) Measurement of the outcome ✅<br>5) Selection of the reported result ✅	✅
TEMPO-2	2024	Open-label	Blinded	1) Randomization process ✅<br>2) Deviations from intended interventions ✅<br>3) Missing outcome data ✅<br>4) Measurement of the outcome ✅<br>5) Selection of the reported result ✅	✅
TRUST	2025	Open-label	Blinded	1) Randomization process ✅<br>2) Deviations from intended interventions ✅<br>3) Missing outcome data ✅<br>4) Measurement of the outcome ✅<br>5) Selection of the reported result ✅	✅

sTable 4. The Grading of Recommendations, Assessment, Development, and Evaluation (GRADE) system for the outcomes of interest

Outcome category	Outcome	No. of participants (Studies)	Study design	Risk of bias	Inconsistency	Indirectness	Imprecision	Publication bias [a]	Effect (OR)	Certainty (GRADE)
Primary effectiveness	mRS 0-1 at 90 days	4360 (5 studies)	RCT	Low	None	Uncertain	Narrow CI	Unlikely	OR 0.85 (95% CI 0.72, 1.00)	High
Other effectiveness	mRS 0-2 at 90 days	4360 (5 studies)	RCT	Low	None	Uncertain	Narrow CI	Unlikely	OR 0.85 (95% CI 0.63, 1.13)	High
	Symptomatic intracranial hemorrhage within 36 hours	4365 (5 studies)	RCT	Low	None	Uncertain	Wide CI	Unlikely	OR 4.70 (95% CI 1.76, 12.52)	High
	All-cause mortality at 90 days	4361 (5 studies)	RCT	Low	Yes	Uncertain	Narrow CI	Unlikely	OR 1.62 (95% CI 0.69, 3.79)	Moderate

CI = confidence interval; HR = hazard ratio; RCT = randomized controlled trial.

[a] It has been shown that tests for publication bias lack sufficient power for the meta-analyses with less than ten studies. Therefore, formal statistical analyses for publication bias were not conducted due to a limited number of included RCTs. However, considering our comprehensive search and the type of eligible studies for this meta-analysis (i.e., RCTs that require a prespecified protocol and registration in publicly available databases), we considered publication bias unlikely to affect our pooled estimates.

sTable 5. Sensitivity analyses by excluding one trial at a time from the pooled estimates.

**Excluded trial**	**Pooled OR (95% CI) using random effect**	**P-value for overall effect**	**Heterogeneity**
**mRS 0-1**			
ARAMIS	0.86 (0.72, 1.03)	0.10	I2 = 0%; P = 0.82
PRISMS	0.85 (0.71, 1.02)	0.08	I2 = 0%; P = 0.73
PUMICE	0.88 (0.72, 1.07)	0.19	I2 = 0%; P = 0.85
TEMPO-2	0.82 (0.67, 1.01)	0.06	I2 = 0%; P = 0.80
TRUST	0.82 (0.68, 1.00)	0.05	I2 = 0%; P = 0.82
**mRS 0-2**			
ARAMIS	0.81 (0.58, 1.13)	0.22	I2 = 46%; P = 0.14
PRISMS	0.88 (0.63, 1.23)	0.45	I2 = 50%; P = 0.11
PUMICE	0.91 (0.62, 1.32)	0.60	I2 = 46%; P = 0.14
TEMPO-2	0.93 (0.65, 1.33)	0.68	I2 = 35%; P = 0.20
TRUST	0.73 (0.57, 0.94)	0.01	I2 = 0.0%; P = 0.65
**Symptomatic intracranial hemorrhage w**ithin 36 hou**rs**			
ARAMIS	5.14 (1.73, 15.24)	0.003	I2 = 0%; P = 0.83
PRISMS	4.19 (1.48, 11.87)	0.007	I2 = 0%; P = 0.90
PUMICE	4.21 (1.48, 11.93)	0.007	I2 = 0%; P = 0.89
TEMPO-2	5.02 (1.42, 17.75)	0.010	I2 = 0%; P = 0.80
TRUST	5.22 (1.76, 15.48)	0.003	I2 = 0%; P = 0.85
**All-cause mortality**			
ARAMIS	1.64 (0.59, 4.54)	0.34	I2 = 69%; P = 0.02
PRISMS	1.56 (0.62, 3.95)	0.35	I2 = 68%; P = 0.02
PUMICE	1.64 (0.46, 5.90)	0.45	I2 = 69%; P = 0. 02
TEMPO-2	1.12 (0.54, 2.35)	0.76	I2 = 28%; P = 0.24
TRUST	2.36 (1.33, 4.19)	0.003	I2 = 0%; P = 0.50
**New vascular events**			
ARAMIS	0.93 (0.64, 1.36)	0.72	I2 = 0%; P = 0.83
PRISMS	0.96 (0.65, 1.43)	0.86	I2 = 0%; P = 0.69
TEMPO-2	0.89 (0.57, 1.39)	0.61	I2 = 0%; P = 0.77
TRUST	1.08 (0.60, 1.97)	0.79	I2 = 0%; P = 0.78

sTable 6. Analyses using random-effect models for the outcomes of interest.

**Outcomes**	**Fixed effect**	
	Pooled OR (95% CI) for IA thrombolysis vs. control	P-value for overall effect
mRS 0-1	0.85 (0.72, 1.00)	0.06
mRS 0-2	0.83 (0.67, 1.03)	0.10
Symptomatic intracranial hemorrhage within 36 hours	5.17 (1.97, 13.55)	<0.001
All-cause mortality	1.62 (1.03, 2.55)	0.04
New vascular events	0.95 (0.65, 1.39)	0.80
